# Recurrence of a Mediastinal Germ-Cell Tumor as a Somatic-Type Malignancy: A Complex Case Report

**DOI:** 10.3390/ijms22179310

**Published:** 2021-08-27

**Authors:** Caroline C. C. Hulsker, Mariëtte E. G. Kranendonk, Thomas F. Eleveld, Ad J. M. Gillis, Cornelis P. van de Ven, Natasha K. A. van Eijkelenburg, Niels P. van der Kaaij, Alida F. W. van der Steeg, Leendert H. J. Looijenga

**Affiliations:** 1 Princess Máxima Center for Pediatric Oncology, 3584 CS Utrecht, The Netherlands; m.e.g.kranendonk-4@prinsesmaximacentrum.nl (M.E.G.K.); t.f.eleveld@prinsesmaximacentrum.nl (T.F.E.); a.j.m.gillis@prinsesmaximacentrum.nl (A.J.M.G.); c.p.vandeven-4@prinsesmaximacentrum.nl (C.P.v.d.V.); n.k.a.vaneijkelenburg-2@prinsesmaximacentrum.nl (N.K.A.v.E.); a.f.w.vandersteeg@prinsesmaximacentrum.nl (A.F.W.v.d.S.); l.looijenga@prinsesmaximacentrum.nl (L.H.J.L.); 2 Department of Pathology, University Medical Center Utrecht, 3584 CX Utrecht, The Netherlands; 3 Department of Cardiothoracic Surgery, University Medical Center Utrecht, 3584 CX Utrecht, The Netherlands; n.p.vanderkaaij-2@umcutrecht.nl

**Keywords:** mediastinal germ-cell tumor, metastasis, molecular diagnostics, clonality, gain 12p, AFP, HCG, microRNA371a-3p, case report

## Abstract

Background and case: An adolescent male presented with a second mediastinal tumor 1.5 years after treatment of a proven malignant germ-cell tumor in that location. The differential diagnosis included a recurrent germ-cell tumor or a non-germ cell malignancy. Serum tumor markers alpha-fetoprotein (AFP) and human chorionic gonadotrophin (HCG) were negative. The first biopsy was not informative, and the second biopsy gave a broad differential diagnosis including secondary non-germ cell malignancy using histology and immunohistochemistry. DNA methylation profiling, RNA sequencing, and targeted microRNA371a-3p profiling was subsequently performed, without a supportive result. After resection of the tumor the definitive diagnosis yielded two secondary non-germ cell malignancies in the form of a leiomyosarcoma and a solitary neuro endocrine carcinoma (NEC). In spite of the differences between the molecular profiles of the initial germ-cell tumor, the leiomyosarcoma and large-cell NEC are clonally related, as determined by the presence of identical chromosomal breakpoints. The copy number profiles suggest an initial polyploidization step, followed by various independent chromosomal gains and losses. This case demonstrates that germ-cell tumors must be evaluated carefully, including molecularly, in which the non-germ cell malignancy is negative for miR-371a-3p, both in tissue as well as in serum, in contrast to the primary tumor. We conclude that the patient presented with a primary type II mediastinal GCT and, a year and a half later, followed by a leiomyosarcoma and a large-cell NEC presenting as two secondary somatic-type malignancies clonally related to the original GCT. Conclusions: Malignant germ-cell tumors are known to recur as a somatic-type malignancy in very rare cases. This case report illustrates the challenges faced in defining the nature and clonality of the secondary somatic-type malignancies.

## 1. Background

Germ-cell tumors (GCTs) represent a highly heterogeneous group of neoplasms [1]. They occur from the neonatal period through to late adulthood and arise at gonadal or extragonadal sites, including the mediastinum, and display a great variety of morphologic elements in pure or mixed forms [2,3]. Overall, malignant GCTs are classified into germinoma-like (including seminoma and (dys)germinoma) and nongerminomatous tumors (yolk sac tumor [YST], choriocarcinoma, embryonal carcinoma [EC] and teratoma). This group of heterogeneous neoplasms is unique in that their developmental potential is, in effect, determined by the latent potency state of their cells of origin, which are reprogrammed to omnipotent/totipotent or pluripotent stem cells [2,4]. Serum tumor markers (STMs) are only reliable when restricted subtypes are represented in the GCT, especially related to AFP and HCG for YST and choriocarcinoma, respectively [5]. Definitive histopathological diagnosis guides further treatment. For gonadal primary sites, initial management entails complete surgical resection regardless of STM status. For extragonadal primary disease, radiological findings combined with STMs may point at a diagnosis, but in case of a negative marker status, a biopsy is required for a histopathological diagnosis [3]. After identification of a malignant GCT and during follow-up, serial radiological imaging to detect possible relapse carries a risk of associated cumulative radiation exposure [6,7]. However, this approach often needs to be included in the clinical management because STMs may not be reliable, as referred to earlier [5]. Recent developments regarding microRNA (especially related to miR371a-3p) quantification for diagnosing and follow-up of malignant GCTs, predominantly studied in testicular primaries, offer a potentially promising improvement in diagnosis and follow-up [8,9]. Mediastinal GCTs account for 2% to 5% of GCTs, and for 50% to 70% of all extragonadal GCTs [10]. Recurrence of disease as growing teratoma syndrome or somatic-type malignancy is a rare but well-known and challenging clinical phenomenon.

Herein we describe a patient with a history of an initially STM-positive mixed-malignant (type II) mediastinal GCT, composed histologically of seminoma, EC, YST and teratoma. It was incompletely removed, after which the patient was treated with chemotherapy. A second tumor was identified a year and a half later and was STM-negative, including miR371a-3p, in which multiple CT-guided biopsies failed to yield a definitive diagnosis. Molecular analysis did not support a diagnosis of a recurrent GCT either. Upon gross total resection, the tumor was diagnosed as two secondary somatic-type malignancies with the histology of leiomyosarcoma and a large-cell NEC, demonstrated to be clonally related to the primary tumor, in spite of heterogeneous chromosomal copy number profiles but a common breakpoint.

## 2. Case

### 2.1. Clinical Description and Histological Findings

**Figure 1:**
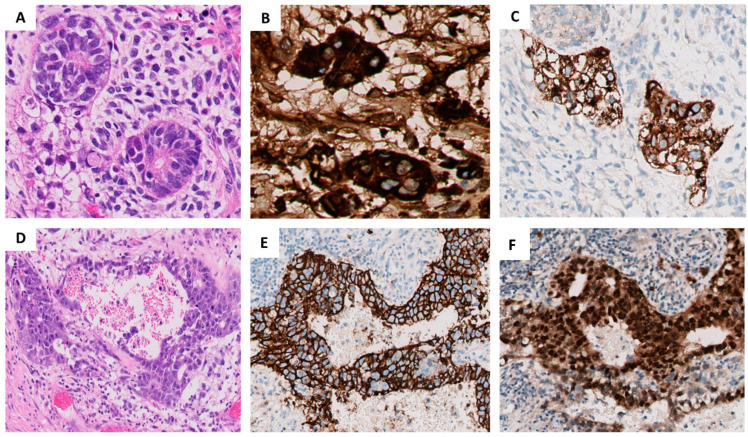
Hematoxylin-eosin (H and E) stains and immunohistochemistry of the primary mediastinal germ-cell tumor: (**A**) H and E of the YST component showing a prominent characteristic Schiller-Duval body (magnification 20×); (**B**) AFP immunostaining showing positive staining in the YST component (magnification 20×); (**C**) Glypican-3 immunostaining showing positive staining in the YST component (magnification 20×); (**D**) H and E of the EC (magnification 10×); and respective immunostaining for (**E**) CD30 and (**F**) OCT3/4 (magnification 10×).

A 16-year-old male presented with a primary tumor in the anterior mediastinum. Thorough work-up showed no other tumor localizations. The tumor was resected from the anterior mediastinum along with a wedge resection of the left upper lobe, partial resection of the pericardium and thymus due to direct tumor invasion, and dissecting of the tumor off the phrenic nerve, which was encased by it. The histology showed a 10 cm diameter mixed malignant GCT, post-pubertal type (Type II), consisting of mature and immature teratoma (40%), seminoma (30%), EC (20%), and YST (10%) (Figure 1). The tumor showed growth with continuous spread into lung, thymus, pericardium, adjacent lymph nodes, and resection margins. Also, parts of tumor that were dissected off the phrenic nerve showed immature teratoma and EC, comprising an incompletely resected, stage III mediastinal-malignant GCT. Molecular analysis was not done at this stage. At initial presentation, serum AFP and HCG were elevated, at 1400 µg/L (reference range 0.0–9.0 µg/L) and 120 IU/L (reference range 0.0–3.0 IU/L), respectively.

**Figure 2:**
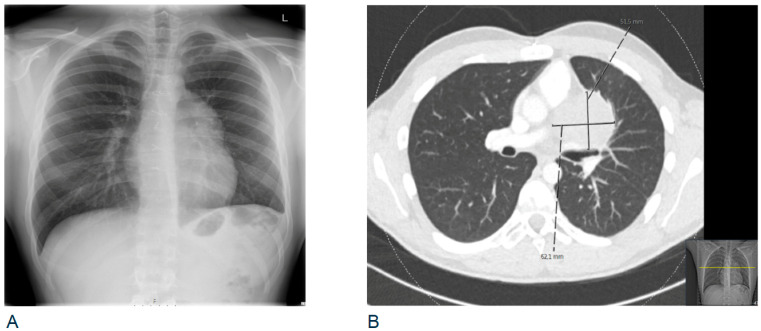
(**A**) Follow-up chest X-ray showing a broadened aorto-pulmonary window a year and a half after resection of malignant mediastinal GCT; (**B**) CT scan confirming recurrent mass in the anterior mediastinum.

The patient was treated with adjuvant chemotherapy, consisting of four cycles of cisplatin, etoposide, and ifosfamide (PEI), and achieved complete remission. During a follow-up chest X-ray, a year and a half after resection, a broadened aorto-pulmonary window was seen (Figure 2), which prompted evaluation by a CT scan. This showed a recurrent mediastinal mass, as well as lung and lymph node metastases, without concomitant elevation of serum AFP or HCG. 

Therefore, six CT-guided Tru-Cut biopsies were taken, showing mostly necrotic tissue, with only fibroblasts of reactive connective tissue recognizable in the vital parts of tissue. A repeat CT-guided procedure yielded two Tru-Cut biopsies, of which the histology showed stromal tissue of unsure etiology, morphologically most fitting with recurrence of the original GCT, based on its resemblance to the stromal component of the original tumor. Immunohistochemistry was performed and was found to be negative for ALK, CD31, or ERG. There was dubious expression of ROS and partial expression of SMA, S100, and SOX10. There was positive expression of INI1. This immune profile was not directional, nor could recurrence of the stromal part of the primary teratoma be confirmed by immunohistochemistry. DNA methylation profiling was performed, in an attempt to be guided towards a more definitive diagnosis, based on the soft tissue classifier, but was not found to be informative (data not shown).

Next, thoracoscopic biopsies were attempted, but the procedure was abandoned because of a completely inaccessible left hemithorax due to previous surgery and tumor mass. Based on these unequivocal pathology results, a wait-and-scan policy was adopted. The lesion showed slow progression and liquid biopsies were obtained. 

As the clinical condition of the patient deteriorated, possibly due to pressure of the mass on the left pulmonary artery and left upper pulmonary vein, and as tissue for histopathological evaluation was needed, tumor debulking was planned. This was performed via a combined sternotomy and left thoracotomy. A left upper lobe resection along with resection of the phrenic nerve and reconstruction of the left pulmonary artery was carried out. 

Macroscopic evaluation showed irradically resected margins, tumor ingrowth into the lung tissue, and three separate pulmonary metastases. Microscopy showed a heterogeneous tumor, mostly consisting of the proliferation of cells resembling smooth muscle cells, together with strongly atypical spindle cells, mixed with myxoid stroma. There were some spindling patterns of the cells, but mostly there was no recognizable cell pattern. The nuclei were pleomorphic and hyperchomatic, including polynucleation. Mitotic activity varied, with areas containing five mitotic figures per 2 mm^2^. Immunohistochemical analyses demonstrated a positive staining for alpha-SMA and caldesmon. Glypican-3 stained only weakly, and OCT4 showed cytoplasmic stain, rather than nuclear, and was therefore considered negative; therefore, again, a GCT identity could not be confirmed. Further negative staining was found for PLAP, SALL4, AFP, CD34, desmin, myo-D1, myogenin, and CK AE1/3 staining, therefore also not confirming a GCT identity. There were some areas of cartilage with strongly atypical cells, in addition to focal bone formation. It was concluded that the recurrent tumor was a recurrent malignant GCT, 12.5 cm diameter, nearly completely overgrown by a secondary somatic-type non-germ cell malignancy, classified as a leiomyosarcoma. In the pre-existent lung parenchyma, there were subpleural and pulmonary metastases. Last, there was another isolated pulmonary lesion (5.8 mm in diameter) composed of clusters of atypical, neuro-endocrine-looking cells strongly positive for CK AE1/3 staining and partially positive for chromogranin A staining, classified as large-cell neuro-endocrine carcinoma (NEC) (based on the high proliferation index (Ki67 labelling 65%) and mitotic frequency of 22/10HPF).

### 2.2. miRNA

**Figure 3:**
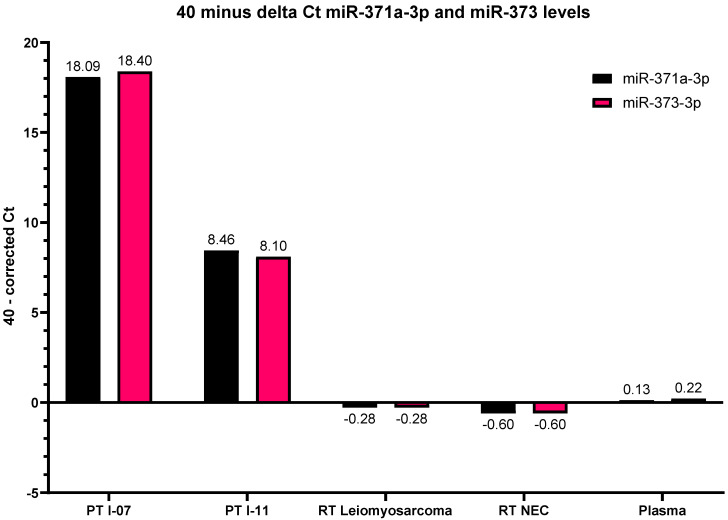
High levels of embryonic miRNAs 371a-3p and 373-3p in the primary tumor sections PT I-07 and intermediate levels thereof in PT I-11, due to the high percentage of teratoma (known to be negative for these miRNAs), and negative results for both the leiomyosarcoma and NEC elements, as well as matched plasma.

To evaluate the miRNA profiles of the different tumors, total RNA was isolated from two representative FFPE-blocks of the primary tumor (PT I-07 and PT I-11), representing the seminoma (40%), EC (20%), YST (10%), and mature and immature teratoma (30%), and mature and immature teratoma (85%) with small regions of seminoma (10%) and EC (5%), respectively. No serum or plasma was available from the time of primary diagnosis. In addition, RNA was isolated from both the recurrent leiomyosarcoma and NEC lesion. High levels of the embryonic miR371a-3p and 373-3p were detected in PT I-07 and intermediate levels in PT I-11, due to the high percentage of teratoma negativity for these miRNAs (Figure 3). These specific miRNAs could neither be detected in the recurrent tumors, nor in the plasma sample taken at the time of recurrence.

**Figure 4:**
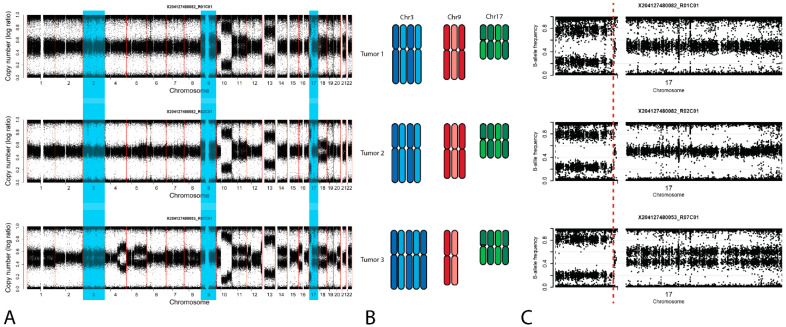
Representative examples of the molecular analyses performed using SNP profiling. Tumor 1 = primary GCT; Tumor 2 = leiomyosarcoma; Tumor 3 = NEC; (**A**) Total SNP profile showing relative loss of chromosome 9, 11, 13 and 18, as well as 17p, and a relative gain of 21, (**B**) copy-number (log ratio) representation of the different histological elements; (**B**) schematic representation of the explanatory constitution of chromosomes 3, 9, and 17, assuming an early polyploidization step; (**C**) B-allele frequency plot of chromosome 17, demonstrating the identical 17p11.2 breakpoint in all histologies.

Molecular findings: Because of the non-informativity of the methylation profile, a SNP array was performed on the tissue of the primary GCT, as well as the resected leiomyosarcoma and the large-cell NEC. The generated profiles of the original GCT and the leiomyosarcoma showed partly similar patterns, with relative loss of chromosome 9, 11, 13, and 18, and copy-number-neutral loss of heterozygosity of chromosome 17p (Figure 3). Neither tumor showed a gain of chromosome 12, including the short arm, which is characteristic for GCTs. The molecular SNP profile of the large-cell NEC was different from the GCT and leiomyosarcoma, and was initially considered as clonally unrelated, in part based on the different pattern of chromosome 9, which was considered to be lost in the GCT and leiomyosarcoma but unchanged in the NEC (Figure 4A). However, closer evaluation resulted in an adjustment of this first conclusion, based on the following aspects The different pattern of chromosome 9 can be explained assuming a polyploidization step in the early pathogenesis, and subsequent loss of either one (GCT and leiomyosarcoma) or two (NEC) of the parental chromosomes, which corresponds with the observed bi-allele frequencies (Figure 4A,B). This clonality was confirmed by the identification of a specific breakpoint in cytoband 17p11.2, present in all histologies (Figure 4C). This, in fact, proves that all three histological elements are clonally related. In summary, we concluded that the patient presented with a type II mediastinal GCT and, a year and a half later, a leiomyosarcoma and a large-cell NEC presenting as two, secondary somatic-type malignancies clonally related to the original GCT. 

The patient received 25 × 2 Gray radiotherapy on the remaining lesions, including the left lower lobe of the lung. The clinical course was complicated by esophagitis and long-term hoarseness due to a left-sided laryngeal paresis. The patient remains in remission twenty months postoperatively. 

## 3. Discussion

The mediastinal subgroup of GCTs accounts for approximately 4% of all pediatric—including adolescent—GCTs [11,12]. Most studies of malignant mediastinal GCTs in children and adolescents have demonstrated an inferior prognosis, as compared with their gonadal counterparts [13,14,15]. This assessment may be partly attributed to the studies on adolescent and adult mediastinal nonseminomatous GCTs, which reported a particularly poor prognosis [16]. Metastasized or relapsed mediastinal GCT carries a particularly poor prognosis, and follow-up consists of the imaging and evaluation of STMs. The latter are only informative when restricted histological subtypes are represented in the GCTs, particularly YST and choriocarcinoma [5]. Recent years have seen the emergence of embryonic miRNAs, especially the miR371-3 family, as a new generation of biomarkers in diagnosis and follow-up of malignant GCTs, offering a potentially promising improvement [5]. These short, non-protein coding RNAs are highly stable and well suited for disease diagnosis and monitoring, and are present in all malignant GCT components, with the consistent exception of teratoma, regardless of patient age, anatomical site of disease or histological subtype [6,17,18]. 

The case report presented here conveys a number of important messages. First, the overall chromosomal constitution of the primary GCT, being a proven Type-II variant [2] composed of seminoma, EC, YST, and teratoma—and, as such, by definition malignant—did not show a representative GCT chromosomal copy-number pattern. No gain of the short arm of chromosome 12, being characteristic for most Type II GCTs [19] was present, while it contained a relative loss of chromosomes 9, 11, 13, 17p, and 18, and a relative gain of chromosome 21. miR371a-3p and 373 expression, as determined in the tumor itself, is in line with the classification as a Type II GCT [8,9]. However, no blood samples from the moment of initial diagnosis were available for evaluation, but would be expected to be positive for these miRNAs, based on the profound expression in the tumor. None of these miRNAs were found to be elevated in serum at the moment of recurrence, 1.5 years after the primary diagnosis and chemotherapy treatment. This negative finding, confirmed in the tumor sample as well, is explained by the nature of the recurrent tumor, being diagnosed as a somatic-type non-GCT malignancy with the histology of a leiomyosarcoma. In addition, the large-cell NEC component identified was evaluated separately and also found to be negative for these miRNAs. The molecular profile data was initially interpreted as supportive for a clonal relationship between the primary Type II GCT and the leiomyosarcoma and an independent origin of the large-cell NEC. However, this was adjusted based on further detailed evaluation, in which an early polyploidization step, i.e., before the actual clinical presentation of the primary GCT, was considered. Based on this, a clonal relationship between the primary GCT, the leiomyosarcoma and the large-cell NEC was concluded, proven based on identification of a common and identical 17p11.2 (non-centromeric) breakpoint In fact, the different SNP patterns of the chromosomes 3 and 9, initially considered as proof for non-clonality, were explained by a single chromosomal gain and loss (of one but different copy of the parental), respectively. 

It can be hypothesized that the teratoma element, as present in the primary GCT, remained after chemotherapy because of an incomplete resection, progressing to the recurrent leiomyosarcoma and NEC. Currently, no clinically informative STMs are available for non-germ cell malignancies, which is a major limitation in their management to date. 

## 4. Materials & Methods

### 4.1. Immunohistochemistry

Immunohistochemical stainings using antibodies against alpha-1-fetoprotein (Thermo RB-365-A, 1:500), glypican 3 (Cellmarque 1G12, 1:80), CD30 (DAKO M0751, 1:160) and OCT4 (Cellmarque MRQ-10, Ventana RTU) were performed on FFPE slides in Ventana Benchmark Ultra, according to the diagnostic procedure protocols used at the pathology department of the University Medical Center in Utrecht, The Netherlands. 

### 4.2. microRNA Profiling of Plasma and Tumors

miRNAs were isolated from 50 µL plasma, in duplicate, using target-specific anti-miR magnetic beads, as reported before [20]. In short, a KingFisher Flex robot with TaqMan^®^ miRNA ABC Purification Kit Panel A was used to isolate miRNAs. A non-human spike-in ath-miR159a was added to the serum for the quality control of targeted miRNA isolation and cDNA generation. TCam2 seminoma cell-line was included as a positive control. Purified miRs were reverse-transcribed, using TaqMan Micro RNA assays ath-miR159a (000338), hsa-miR30b-5p (000602), hsa-miR371a-3p (002124), and hsa-miR373-3p (000561). To increase sensitivity and specificity, a 12-cycle pre-amplification step was included. For normalization, hsa-miR30b-5p was used as described.

RNA isolation of the primary and recurrent tumor samples was performed from the same FFPE-blocks as used for diagnostics using TRIzol. Briefly, two 10 µm-thick sections were cut and placed into a sterile microfuge tube, deparaffinized, washed with ethanol, and pellets were dissolved in 1 mL TRIzol. The remainder of the protocol was carried out using the manufacturer’s instruction. The concentration and purity of the RNA-samples were assessed using the NanoDrop spectrophotometer. Twenty ng of RNA were reverse transcribed using TaqMan Micro RNA assays RNU48 (001006), hsa-miR371a-3p (002124), and hsa-miR373-3p (000561). For normalization, RNU48 was used.

miRNA levels were detected on a QuantStudio 12K Flex machine. All devices and Kits were purchased from Thermo Fisher Scientific, Bleiswijk, the Netherlands.

## 5. Conclusions 

Malignant germ-cell tumors are known to recur as a somatic-type malignancy in very rare cases. 

This case report illustrates the multiple challenges faced in defining the nature of the secondary somatic-type malignancies, classifiable as leiomyosarcoma and large-cell NEC, 1.5 years after the primary mixed malignant mediastinal GCT.
